# Demonstration of a Very Inexpensive, Turbidimetric, Real-Time, RT-LAMP Detection Platform Using Shrimp Laem-Singh Virus (LSNV) as a Model

**DOI:** 10.1371/journal.pone.0108047

**Published:** 2014-09-25

**Authors:** Narong Arunrut, Rungkarn Suebsing, Boonsirm Withyachumnarnkul, Wansika Kiatpathomchai

**Affiliations:** 1 Center of Excellence for Shrimp Molecular Biology and Biotechnology (CENTEX Shrimp), Faculty of Science, Mahidol University, Bangkok, Thailand; 2 National Center for Genetic Engineering and Biotechnology (BIOTEC), National Science and Technology Development Agency, Pathum Thani, Thailand; 3 Department of Anatomy, Faculty of Science, Mahidol University, Bangkok, Thailand; 4 Aquatic Animal Biotechnology Research Center, Faculty of Science and Industrial Technology, Prince of Songkla University, Surat Thani Campus, Surat Thani, Thailand; University Hospital San Giovanni Battista di Torino, Italy

## Abstract

Rapid and accurate detection of pathogens under field laboratory conditions is necessary for effective control of veterinary pathogens. Here we describe a prototype, portable, pathogen detection device developed for single tube, real-time, reverse transcription, loop-mediated isothermal amplification (RT-LAMP) using Laem-Singh virus (LSNV) as a model. LSNV is an RNA virus and a component cause of growth retardation in black tiger shrimp. We chose its RNA-dependent RNA polymerase (RdRp) gene as the target for our tests. The basis for detection was measurement of turbidity arising from formation of a white, insoluble magnesium pyrophosphate precipitate byproduct upon amplification of the RdRp target sequence from 100 ng template RNA extracted from shrimp. The measurement device consisted of a heating block to maintain constant temperature in the RT-LAMP reaction for 8 Eppindorf sample tubes, a light-emitting diode (LED) light source providing red light emission at 650 nm wavelength to pass through sample tubes, a light dependent resistance (LDR) photo-detector and a software program to report turbidity events and could potentially be marketed for under US$3000. The device was connected to a computer to display real-time results in a variety of formats. The optimized protocol for LSNV detection consisted of incubation of the sample tubes at 65°C for 1 h during which turbidity was continuously measured, and quantitative results could be obtained by reaction time measurement. The sensitivity of detection was comparable to that of conventional nested RT-PCR and there was no cross reaction with other common shrimp viruses. The device was used for quantitative measurement of relative copy numbers of LSNV RdRp in 8 shrimp tissues and they were found to be highest in the gills followed in order by the lymphoid organ and hemolymph (p≤0.05). This platform can be easily adapted for detection of other pathogens under field laboratory settings.

## Introduction

Rapid and accurate detection of pathogens under field laboratory conditions is necessary to effectively control pathogens of terrestrial and aquatic animals. Laem-Singh virus (LSNV) is a positive-sense single-stranded RNA (ssRNA) virus of shrimp that was first discovered from shotgun cloning of nucleic acids isolated from growth-retarded *Penaeus (Penaeus) monodon* from Laem-Singh district of Chantaburi province in Thailand in 2006 [1]. The RNA-dependent RNA polymerase (RdRp) of LSNV shows significant deduced amino acid sequence correspondence to viruses in the family *Leuteoviridae* that comprises mostly insect-borne plant viruses [1]–[3]. By transmission electron microscopy (TEM), LSNV was found to be localized in the lymphoid organ (LO) tissue, gills, haemocytes, brain, eyestalks, optic lobes and ventral nerve cord from farmed shrimp exhibiting monodon slow growth syndrome (MSGS) [4]. Although the preliminary studies suggested that LSNV was not the cause of retarded growth [1], [4], subsequent study revealed that it was specifically associated with retinopathy in stunted black tiger shrimp in MSGS ponds and most likely constituted a necessary but not sufficient cause of MSGS [5]. Later, LSNV was found to be associated with an integrase containing element, present together with LSNV in eyes and lymphoid organs of stunted *P. monodon* [6].

Even though the detailed etiology of MSGS is still uncertain, LSNV is considered to be a component cause of the disease, and it has therefore been placed on the list of specific pathogens to be eliminated from specific pathogen free (SPF) stocks of domesticated *P.monodon* [7]. For the diagnosis of LSNV, traditional RT-PCR and nested RT-PCR methods have been developed to screen broodstock and PL [1], [8], but their use is limited due to constraints related to cost of required equipment and need for highly trained personnel. A more field-friendly method was developed for detection by reverse transcription loop-mediated isothermal amplification (RT-LAMP) followed by hybridization with a specific FITC-labeled probe and visualization using a lateral flow dipstick (LFD) format [9]. However, that method still risked contamination due to the necessity of opening the reaction tube for the probe addition step, and it could not be used to determine viral loads (i.e., severity of LSNV infections).

As LAMP reactions proceed, pyrophosphate ions are released from the deoxyribonucleotide triphosphate (dNTP) reagents consumed during nucleic acid polymerization and these react with magnesium ions in the reaction mix to produce a white, insoluble magnesium pyrophosphate precipitate. This product results in progressively increasing turbidity of the reaction solution [10]. Since the change in turbidity is difficult to quantify by the unaided eye, it may not be clearly visible in the case of a slightly positive reactions [11]. However, this limitation can be overcome by using a turbidimeter, and a real-time turbidimeter has been applied for measuring turbidity of multiple samples instantaneously [12]–[15].

In the present study, a relatively unsophisticated portable device that combined a heating block to support RT-LAMP with a turbidimeter to continuously measure magnesium pyrophosphate precipitate was developed for LSNV detection and quantification under field laboratory conditions. It was also used to determine the number of copies of the LSNV RdRp target in various organs of infected shrimp, providing information on the severity of infection.

## Materials and Methods

### LAMP-Turbidimeter

**Figure 1:**
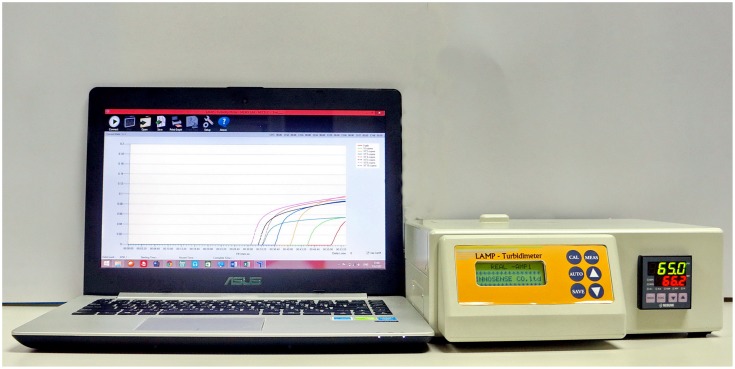
Overview of the LAMP-Turbidimeter measurement system.

The LAMP-turbidimeter allowed for multi-channel turbidity measurements based on spectroscopic assessment of the magnesium pyrophosphate byproduct of LAMP reactions. It consisted of a heating block unit (supporting 8 commercial 0.2 ml thin wall Eppindorf tubes) that maintained a constant temperature during the LAMP reaction, a light-emitting diode (LED) light source providing red light emission at 650 nm wavelength, a light dependent resistance (LDR) photo-detector and a software program to continually (1 second intervals) report turbidity [11]. For measurement of turbidity, light from the LED that passed through reaction tubes to the LDR photo-detector and its signals were converted into output as measureable real-time signals that were sent to a computer for presentation in a variety of formats on a computer screen. The turbidimeter itself has a front-facing LCD screen for calibration and an LED screen to show heating-block temperature. A picture showing the LAMP-turbidimeter measurement system is shown in Fig. 1.

### Shrimp samples

Since the gross signs of LSNV infection are retarded growth that may also arise from many other causes, the only method for easy detection is RT-PCR. To obtain specimens for this work, broodstock from a family of black tiger shrimp (*P.monodon*) with a known history of LSNV infection [16] were collected from the Shrimp Genetic Improvement Center (SGIC) in Surat Thani Province, Thailand and screened for LSNV infection by RT-PCR. Positive specimens were used as a source of LSNV RNA template.

### RNA extraction and *in vitro* RNA transcription

Total RNA was extracted from gills of black tiger shrimp infected with LSNV using a simple and rapid method with 2 M guanidine thiocyanate (GuSCN), and it was used directly for LAMP reactions [9], [17], [18]. A plasmid containing a 588-bp fragment of the LSNV RNA dependent RNA polymerase (RdRp) (amplified using primers LSNVF1 5′-TTC TCC CGA GTG GTC AGG TTTA-3′ and LSNVR1 5′-CCA GAA ACG TAT TGG CAC ACG-3′) was linearized by *Xho*I restriction enzyme (New England Biolabs, USA) at 37°C for 3 h and purified by NucleoSpin Gel and PCR Clean-Up Kit (Macherey-Nagel, Germany) according to the manufacturer’s protocols. An *Xho*I enzyme-digested product was used for RNA *in vitro* transcription by the RiboMAX Large scale RNA production system-T7 kit (Promega, USA) following the manufacturer’s protocol. The quantity of total RNA and *in vitro* RNA transcripts were analyzed by spectrophotometer and subjected to ten-fold serial dilution of 50 ng to 5 fg equivalent to 0.5×10^6^ to 1 copies of the LSNV target per reaction vial, respectively. The RT-LAMP and nested RT-PCR reactions were carried out using 2 µl of RNA template solution.

### Real-time RT-LAMP assays for LSNV

RT-LAMP assays for LSNV detection were carried out according to a previously reported protocol with specific LAMP primers based on the published sequence of the LSNV RNA dependent RNA polymerase (RdRp) (GenBank accession no. DQ127905), except that unlabeled primers were used (i.e., no biotin labeling of primers and no FITC labeling) [9] (Table 1). The reaction mixtures (25 µl) contained 2 µM each of inner primers (FIP and BIP) and loop primers (LF and LB), 0.2 µM each of outer primers (F3 and B3), 2 mM of dNTP mix (Thermo Fisher Scientific, USA), 0.1 M betaine (USB corporation, OH, USA), 6 mM MgSO_4_ (Sigma-Aldrich, USA), 8 U of *Bst* DNA polymerase (New England Biolabs, MA, USA), 1x of the supplied buffer, 0.25 U of *AMV* reverse transcriptase (Promega, USA) and the specified amount of RNA template. To prevent evaporation, reactions were overlaid with 15 µl of mineral oil (Sigma-Aldrich, Steinheim, Germany). Temperature optimization was carried out at 60, 63 and 65°C using a portable turbidimeter [11]. Real-time monitoring of the reactions was achieved by measuring turbidity every second by optical density (OD) at 650 nm using a spectrophotometer.

**Table 1:** Primers used for real-time LAMP to detect LSNV and internal control gene (*β*-actin).

Target	Primer name	Position	Sequence (5′–3′)
LSNV	LSNV-F3	101–120	TCATGCTGCATATGCTTGCT
	LSNV-B3	318–299	TGCGATGTGTTTCATGGTGT
	LSNV-FIP	196–176/TTTT/134–151	CGGCTGAGGTAGCTGCTTGAATT TTGTGAGCCCGTGACTCCTA
	LSNV-BIP	214–233/TTTT/284–265	GCGAAGGCAGGGTGCATTGTTTTTGCGCCCTCAAAGTTAAAACC
	LSNV-LF	155–172	TGTCATCACCGCAGGCTA
	LSNV-LB	238–255	AGTGTCGATCGCAAGCTA
Internal control	*β-*actin-F3	669–686	CTTCGAGCAGGAGATGAC
	*β*-actin-B3	880–863	GGTCCTTACGGATGTCCA
	*β*-actin-FIP	764–745/TTTT/705–722	CTCTCGTTGCCGATGGTGATTTTTCTCGCTGGAGAAGTCCTA
	*β*-actin-BIP	783–802/TTTT/842–825	CCTGTTCCAGCCCTCATTCCTTTTTTGTAGGTGGTCTCGTGG

### One-step RT-PCR and nested RT-PCR for LSNV detection

The nested RT-PCR protocol for LSNV detection was modified from previous publications [1], [8]. Total RNA was extracted from the tissue of normal black tiger shrimp or shrimp suspected of LSNV infection and 100 ng was used as template for RT-PCR reactions with primers targeting the LSNV RdRp gene. A Transcriptor One-step RT-PCR kit (Roche, Mannheim, Germany) was used to produce cDNA by reverse transcriptase (RT) for use as the template for PCR with LSNV-20AF and LSNV-20AR primers (Table 2) [1] to generate a 200 bp amplicon in what constituted a 1-step RT-PCR detection protocol. For the second, nested PCR step, a 2 µl portion of the 1-step RT-PCR reaction solution was used as the template in a second reaction with LSNVn-F and LSNVn-R primers (Table 2) to amplify a 140 bp nested amplicon [8].

**Table 2:** Primers used for RT-PCR and nested RT-PCR for detection of LSNV.

Method	Primer name	Sequence (5′–3′)	References
RT-PCR	LSNV-20AF	TTGCCTTCTCCCGAGTGGTC	Sritunyalucksana et al. 2006
	LSNV-20AR	CCGGCTGAGGTAGCTGCTTG	
Nested RT-PCR	LSNVn-F	GCGCAAGAGTTCTCAGGCTT	Prakasha et al. 2007
	LSNVn-R	ATCACCGCAGGCTAATATAG	

### Comparison of real-time RT-LAMP and conventional, nested RT-PCR assays

To compare the sensitivity of the real-time RT-LAMP assay and the conventional, nested RT-PCR assay, two types of RNA template were used. One consisted of ten-fold serial dilutions of total RNA (50 ng to 0.5 fg) extracted from the gill tissues of shrimp infected with LSNV, while the other consisted of *in vitro* LSNV-RNA transcripts (0.5×10^6^ to 1 copies). They were used as templates under the optimized conditions for the real-time RT-LAMP and the nested RT-PCR assays, as described above.

### Specificity of the real-time RT-LAMP assay

Specificity was tested using RNA templates (100 ng) extracted from shrimp infected with non-target pathogens including the RNA viruses yellow-head virus (YHV), Taura syndrome virus (TSV) and infectious myonecrosis virus (IMNV) and the DNA viruses white-spot syndrome virus (WSSV) and infectious hypodermic and haematopoietic necrosis virus (IHHNV). Controls consisted of RNA template extracted from healthy shrimp not infected with those viruses.

### Quantitative real-time RT-LAMP assays

To quantify target LSNV RNA (i.e., mRNA plus genomic RNA) by the real-time RT-LAMP assay, 10-fold serial dilutions of *in vitro* RNA transcripts were employed. The copy numbers of the *in vitro* RNA transcripts were calculated based on the molecular weight and Avogado’s number and were used to construct a standard curve using the portable turbidimeter. The standard curve for the LSNV RdRp gene was generated individually for each set of samples analyzed. The reaction setup was the same as that optimized above, and the reactions were carried out in the portable turbidimeter.

### Establishment of an internal control for quantitative real-time RT-LAMP

As a reference control for our real-time RT-LAMP assays of LSNV RdRp RNA copy numbers in 100 ng samples of shrimp RNA, shrimp *β*-actin mRNA (GenBank accession number JN808449) was chosen as the target because of previous demonstrations that its expression did not vary greatly from tissue to tissue based on copy numbers per unit of total RNA extract [19]. Specific-LAMP primers were designed using the Primer Explorer version 4 software (Eiken Chemical, Japan) (Table. 1). The target fragment of the *β*-actin mRNA was amplified by RT-PCR using F3 and B3 primers, yielding a cDNA product of 212 bp that was inserted into a plasmid using a pGEM T Easy kit (Promega, USA) according to the manufacture’s protocol. The recombinant plasmid was subjected to 10-fold serial dilution and used to prepare a standard curve of concentration of LAMP DNA amplicon versus turbidometer reading. The same RNA extracts used for LSNV quantification were also used as 100 ng templates under the same optimal reaction conditions as the LSNV real-time RT-LAMP with the turbidimeter device to determine *β*-actin copy numbers. To validate the data for LSNV copy numbers, it was necessary that there be no significant difference in mean *β*-actin copy numbers among the tissue types examined.

### LSNV quantification in shrimp organs by real-time RT-LAMP

A total of 10 black tiger shrimp broodstock suspected of LSNV infections were collected from SGIC and their tissues (lymphoid organ, gills, pleopods, hepatopancreas, heart, haemolymph, gonads and stomach tissues) were aseptically removed and subjected to RNA extraction for analysis by real-time RT-LAMP and by conventional nested RT-PCR assays. Raw data for copy numbers were recorded and converted to a percent relative index to account for different levels of overall infection in the 10 shrimp used for each sample. Specifically, the tissue type with the highest copy number for any shrimp specimen was set at the percent relative index 100 and all other tissues for that specimen were adjusted as a proportion of this number, i.e., Relative Index = (Specific tissue number/Highest tissue number)×100. Then mean relative index and standard deviation (SD) were calculated for each tissue in preparation for statistical analysis by ANOVA followed by all pairwise multiple comparisons using the Holm-Sidak method with Sigmastat for Windows 3.5 software. Differences with p≤0.05 were considered to be statistically significant.

## Results and Discussion

### Real-time RT LAMP conditions

**Figure 2:**
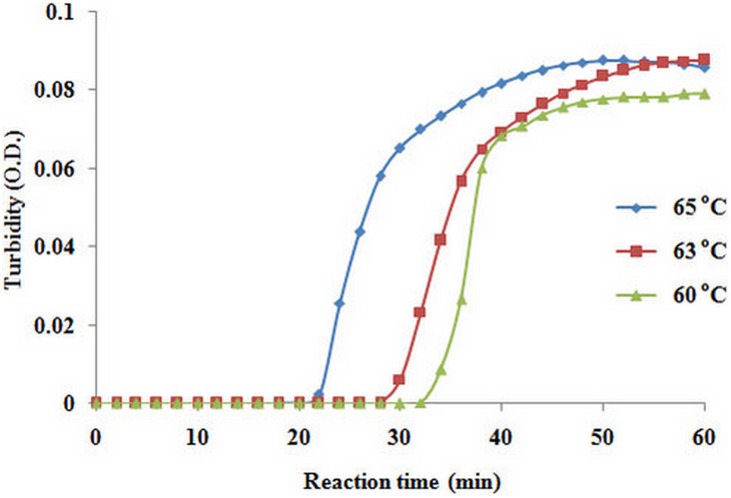
Turbidometer plots for temperature optimization for the LSNV real-time RT-LAMP performed at 60, 63 and 65°C.

Testing real-time RT-LAMP by the portable turbidimeter at 60, 63 and 65°C for 60 min using 100 ng of total RNA as template revealed that RT-LAMP products could be visualized at all tested temperatures. For reaction time, the turbidity was seen first at 22 min and then at 28 and 31 min at 65, 63 and 60°C, respectively (Fig. 2). Therefore, temperature and reaction time at 65°C for 60 min were selected as the optimal conditions for the real-time RT-LAMP assay. The conditions of the real-time RT-LAMP reaction to detect LSNV were similar to those we reported previously for detection of LSNV by RT-LAMP in combination with a lateral flow dipstick [9].

### Sensitivity comparison between real-time RT-LAMP and conventional RT-PCR

**Figure 3:**
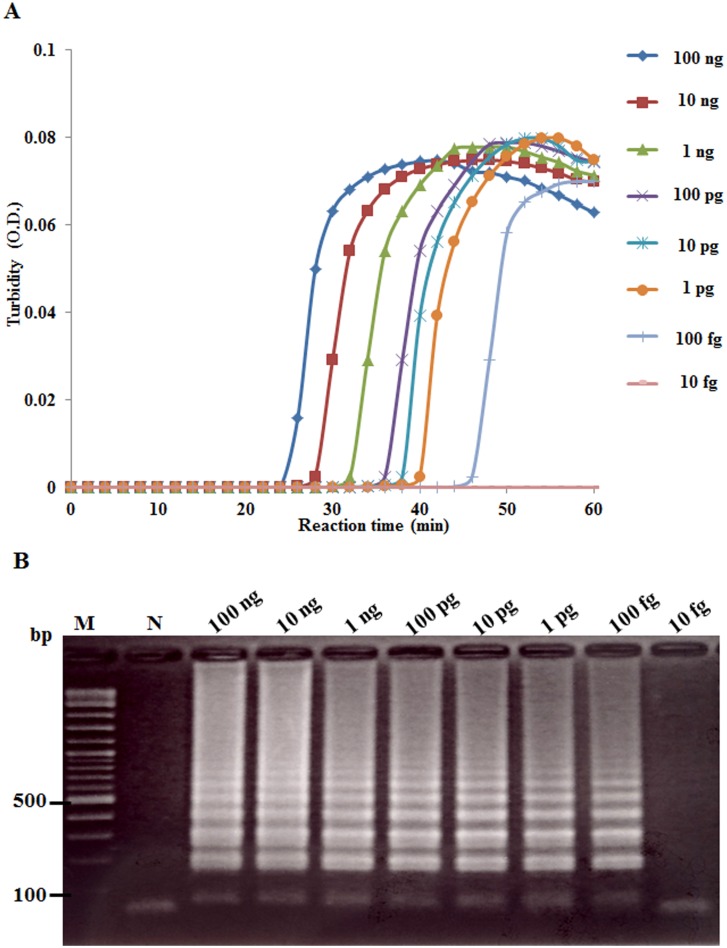
Comparative sensitivity of LSNV real-time RT-LAMP and RT-LAMP followed by gel electrophoresis. (A) Turbidometer plots from assays using 10-fold serial dilutions of RNA extracted from LSNV-infected shrimp. (B) PAGE results using amplicons from the same serially diluted RNA. Results show that both methods could detect LSNV in 100 fg total template RNA. Lane M: 2 log DNA marker and lane N: 100 ng of RNA extracted from normal shrimp.

**Figure 4:**
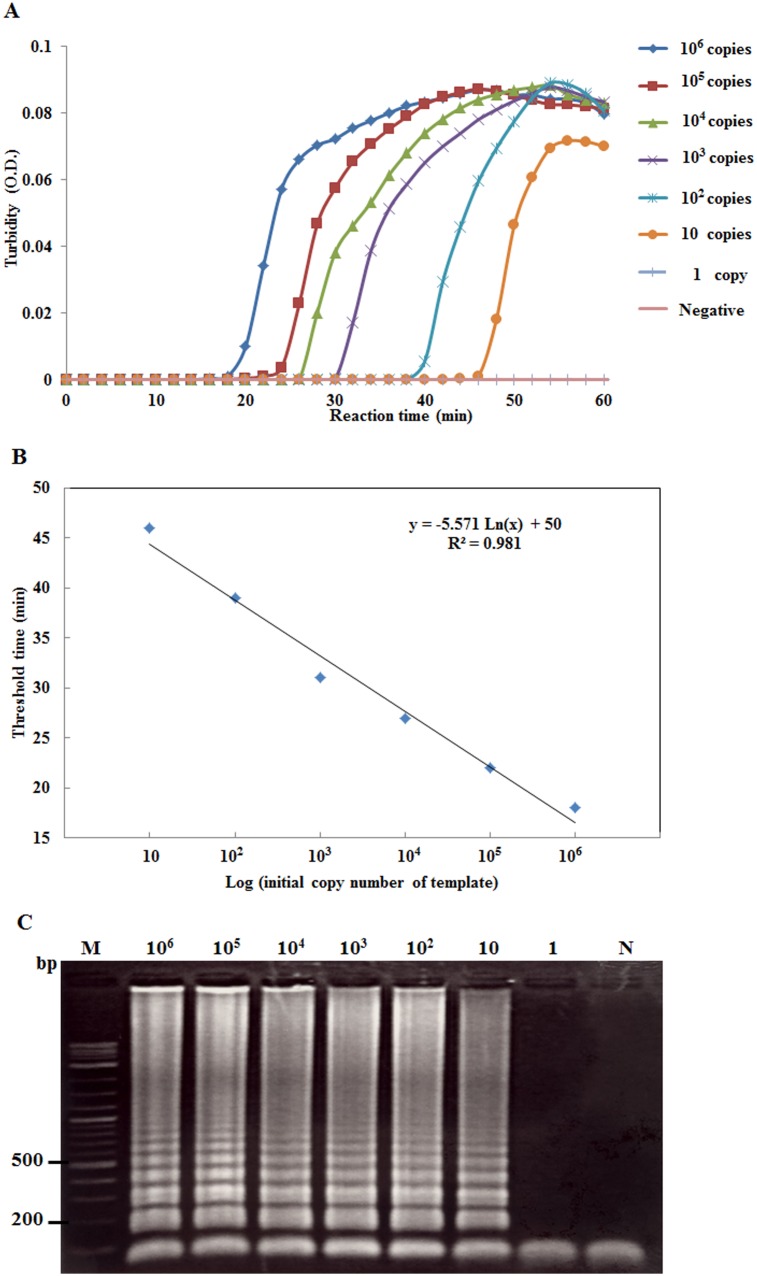
Preparation of a standard curve for real-time LSNV RdRp copy numbers. (A) Time-course, turbidometer plots of OD 650 nm versus copy numbers of *in vitro* LSNV RNA transcripts at concentrations from 1 to 10^6^ copies. (B) The standard curve for RdRp copy number versus threshold time taken from the graph in (A). (C) PAGE results of the real-time RT-LAMP amplicons from (A). Lanes M: 2 log DNA marker and N: negative control.

**Figure 5:**
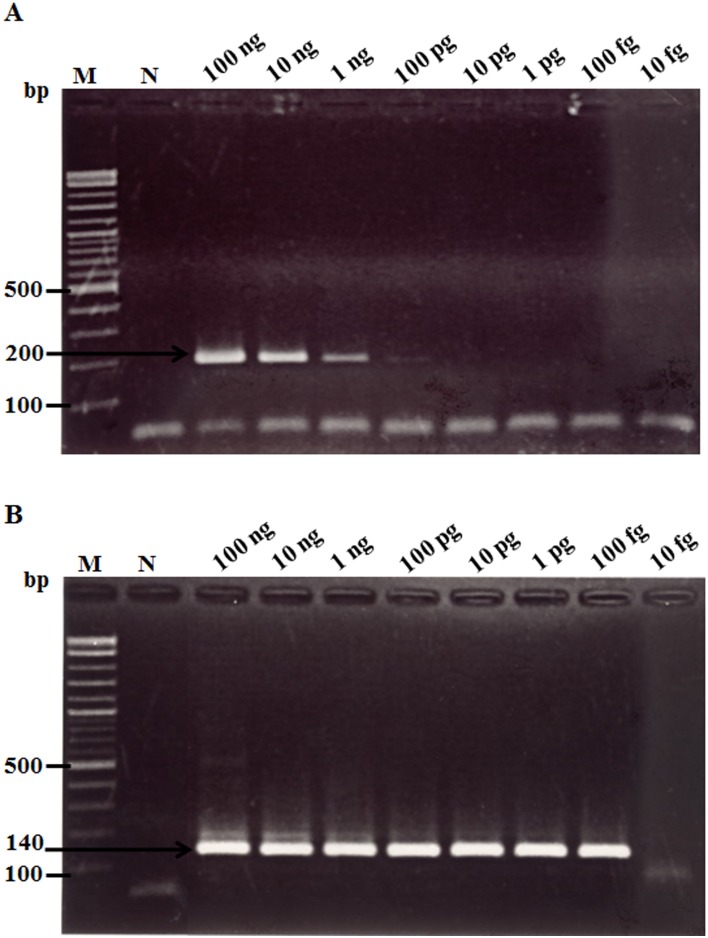
Sensitivity of LSNV detection by 1-step RT-PCR (A) and nested RT-PCR (B). The limit of detection for LSNV for 1-step PCR was 1 ng of total RNA template while that for nested RT-PCR was 100 fg. The latter was the same sensitivity as that for the turbidometer (Fig. 3). Lane M: 2 log DNA marker and lane N: 100 ng of RNA extracted from normal shrimp.

When the real-time RT-LAMP assay with the turbidimeter was used with various RNA template concentrations (i.e., either total RNA extracted from infected shrimp or *in vitro* LSNV RNA transcripts), it was able to detect templates at as little as 100 fg of total RNA (Fig. 3A) and 10 copies of *in vitro* LSNV RNA transcripts (Fig. 4A). This corresponded with the sensitivity of real-time RT-LAMP followed by gel electrophoresis (Fig. 3B, 4C). The RT-PCR and nested RT-PCR methods were able to detect template at 100 pg (Fig. 5A) and at 100 fg (Fig. 5B), respectively. Thus, sensitivity of the real-time RT-LAMP method was the same as that of nested RT-PCR (100 fg), and both were 1000-times more sensitive than traditional one-step RT-PCR detection. The levels of sensitivity for real-time RT-LAMP/turbidimeter detection were similar to those previously reported for RT-LAMP combined with a lateral flow dipstick [9], and for other non-quantitative RT-LAMP methods for detection of shrimp viruses [20]–[22].

### Specificity of real-time RT-LAMP detection

The tests for cross reaction of the real-time RT-LAMP assay with RNA extracts from shrimp infected with other common shrimp viruses (i.e., RNA viruses YHV, TSV and IMNV, and DNA viruses WSSV and IHHNV) and with RNA templates from healthy shrimp gave no positive results, indicating that the real-time RT-LAMP assay was specific for LSNV. As with sensitivity, specificity of our protocol was similar to that previously reported for non-quantitative RT-LAMP for LSNV detection [9], and for detection of other shrimp viruses [20]–[24] by non-quantitative RT-LAMP methods.

### Quantitative real-time RT-LAMP detection of LSNV

**Figure 6:**
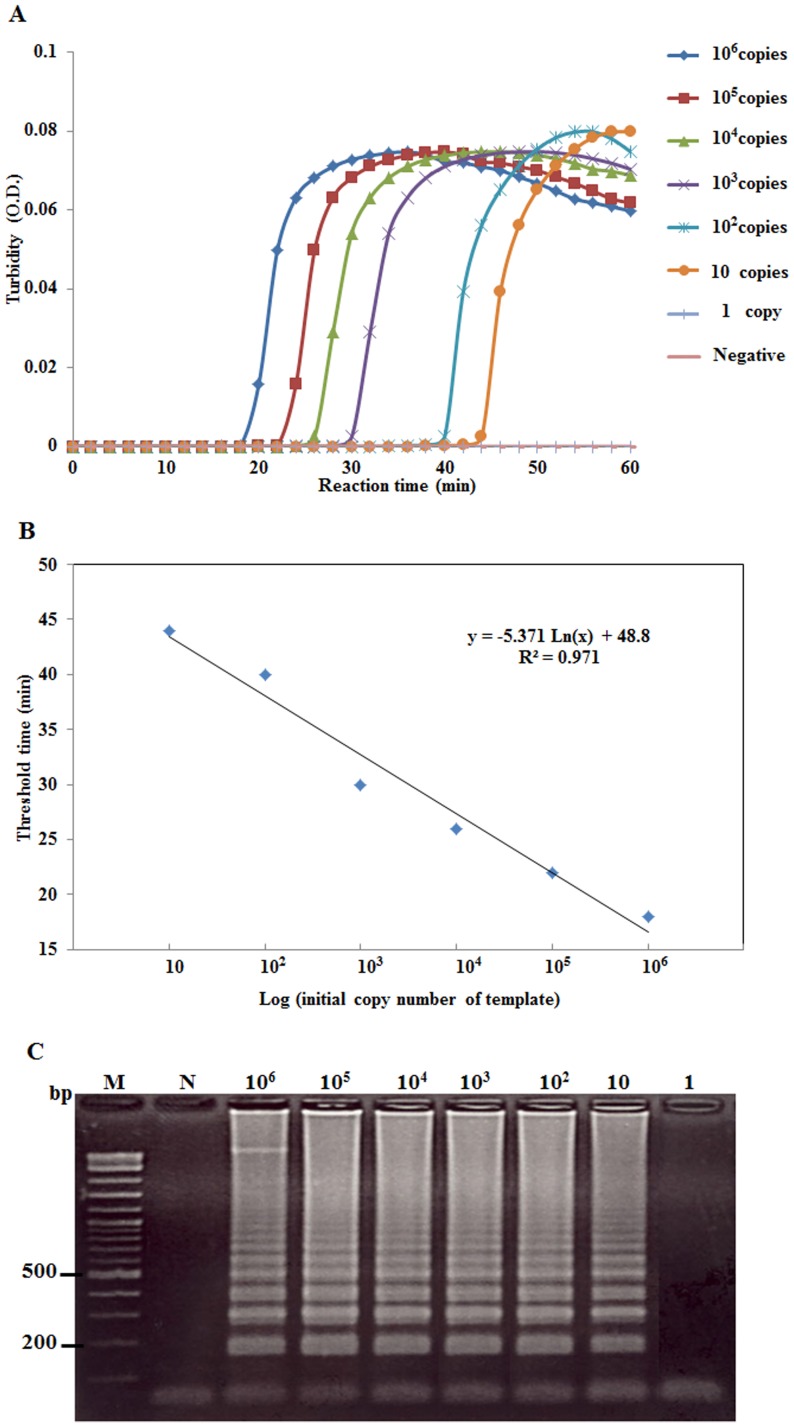
The standard curve for real-time *β*-actin copy numbers. (A) Time-course plots for OD 650 nm versus *β*-actin DNA plasmid at concentrations from 1 to 10^6^ copies. (B) The standard curve for *β*-actin copy numbers versus threshold time taken from the graph in (A). (C) PAGE results from the real-time RT-LAMP reactions shown in (A). Lanes M: 2 log DNA marker and N: negative control.

For the real-time RT-LAMP assay, preparation of a curve of turbidity time (*T_t_*) plotted against log of the initial template quantity using 10-fold serially diluted *in vitro* LSNV RNA transcripts (from 10^6^ to 10 copies per reaction tube) resulted in a line plot with a high correlation coefficient (R^2^ = 0.981) (Fig. 4A–B). The sensitivity was similar to that of real-time RT-LAMP analyzed by agarose gel electrophoresis (AGE) (Fig. 4C). Similarly, real-time RT-LAMP assay using ten-fold serial dilutions (10^6^ to 10 copies) of plasmid DNA containing *β*-actin gene gave a straight line with a high correlation coefficient (R^2^ = 0.971) (Fig. 6A–B) and it showed similar sensitivity to real-time RT-LAMP analyzed by AGE (Fig. 6C). These standard curves were subsequently used for the study of comparative LSNV RdRp copy numbers and *β*-actin copy numbers in organs of infected shrimp. Althlough the copy numbers for *β*-actin could be attributed to mRNA, our method could not distinguish between genomic copies and mRNA copies of the RdRp from LSNV.

### LSNV RdRp copies in shrimp organs determined by real-time RT-LAMP

When 8 tissues each from 10 broodstock specimens were tested by the finalized real-time RT-LAMP method, it was found that all 80 tissues samples from the 10 shrimp were positive for LSNV over a range of 48 to 8.6×10^5^ LSNV copies per 100 ng total RNA. Parallel tests by normal nested RT-PCR were also LSNV positive for all 80 samples while only 56 were positive by the less-sensitive, one-step RT-PCR used, which by comparison to the RT-LAMP results was only capable of detecting LSNV when virus copy numbers were relatively high (i.e., over 1.6×10^3^ copies per 100 ng total RNA).

**Figure 7:**
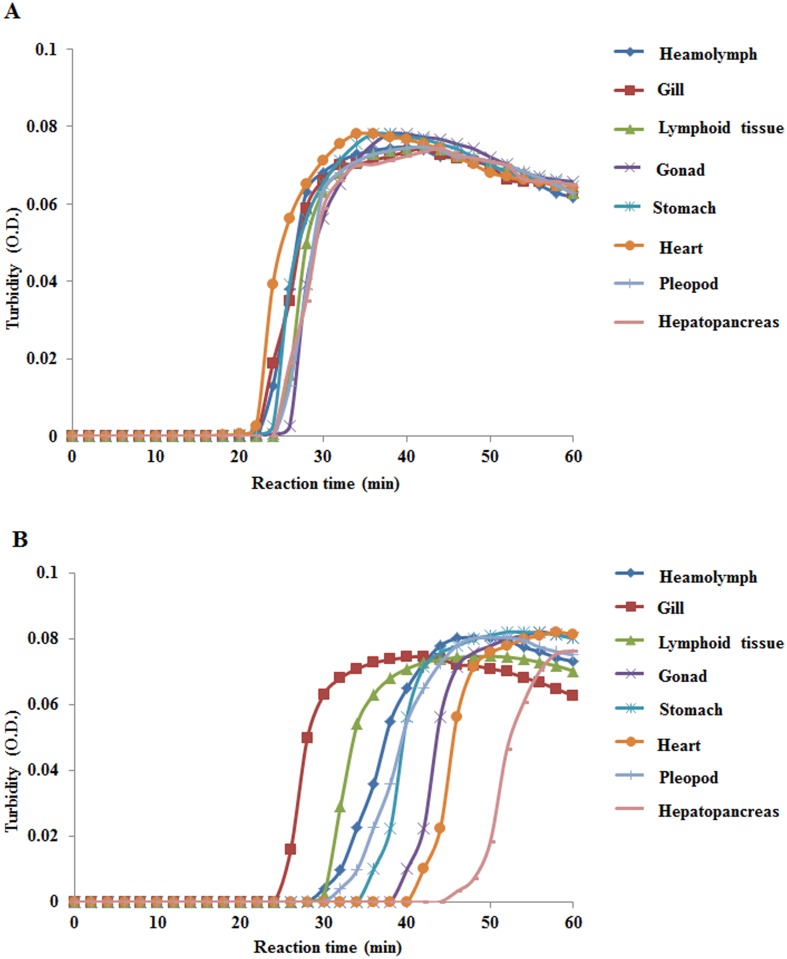
Example of quantitative detection of LSNV RdRp and *β*-actin mRNA in 100 ng total RNA extracts from 8 tissues of a single shrimp specimen infected with LSNV. (A) Relatively uniform quantitative plots for *β*-actin the various tissue extracts. (B) Much more variable plots for LSNV in the same tissue extracts.

For comparison of relative LSNV copy numbers in 100 ng of total RNA extracted from various tissues of LSNV infected shrimp, *β*-actin was chosen as the internal control target gene. Results for individual shrimp (see example for 1 shrimp specimen in Fig. 7A) revealed visually similar real-time RT-LAMP curves (i.e., similar levels of *β*-actin expression) among the various tissues tested. However, an ANOVA test of mean relative copy indices of *β*-actin per 100 ng RNA for 8 tissues in 10 shrimp specimens revealed a significant difference (p = 0.013) among tissues of the 10 shrimp specimens used. A subsequent, all pairwise multiple comparison by the Holm-Sidak method revealed that this ANOVA result arose from a significant difference only between the pair of RNA from gill tissue with RNA from hepatopancreatic tissue (i.e., the difference between the highest and lowest means). When the result for hepatopancreatic tissue was left out of the ANOVA analysis, there was no significant difference in the mean copy numbers among the 7 remaining tissues by a one way ANOVA test (p = 0.180). Thus, we considered that *β*-actin expression was a suitable internal control and that its expression did not vary significantly among the 100 ng RNA templates obtained from the tissues tested. Thus, one way ANOVA analysis of the unadjusted mean relative copy indices for LSNV were used to compare expression of LSNV in 8 tissues from 10 shrimp.

**Figure 8:**
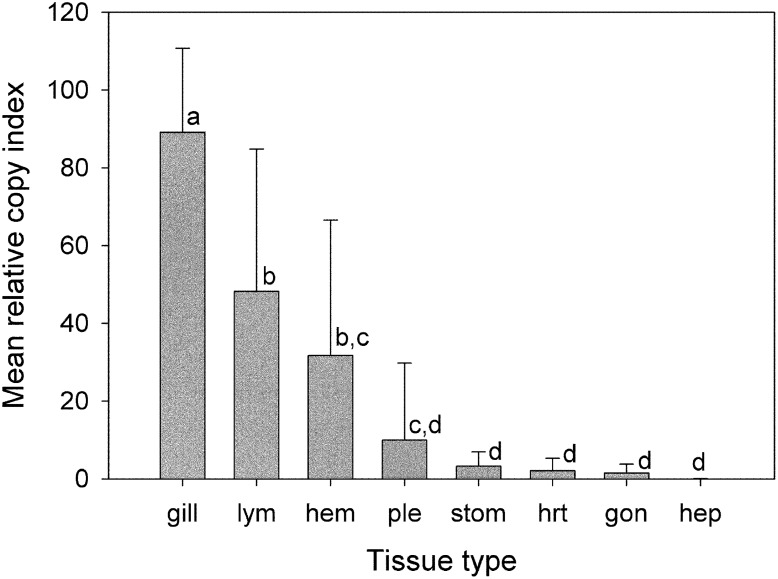
Graph showing a comparison of mean relative copy indices for LSNV RdRp in various shrimp tissues indicated by vertical bars plus standard deviation (SD) markers. Means were obtained by using individual real-time RT-PCR graphs for 8 tissues from 10 individual shrimp, similar to the example shown for one shrimp specimen in Fig. 6A. Mean relative copy indices (bars) marked with any letter in common are not significantly different (p>0.05) while those with no letter in common are significantly different (p≤0.05). lym = lymphoid organ, hem = hemolymph, ple = pleopod, stom = stomach, hrt = heart, gon = gonad, hep = hepatopancreas.

In contrast to the relatively uniform real-time RT-LAMP curves for *β*-actin in various tissues of individual shrimp, those for LSNV were quite variable (see example for one shrimp in Fig. 7B). One way ANOVA revealed a significant difference (p = 0.001) in the mean relative copy index for LSNV among the 8 tested tissues using the same 100 ng RNA templates used for the *β*-actin analysis. All pairwise multiple comparisons by the Holm-Sidak method revealed that the highest level of LSNV was in gill tissue, followed by the lymphoid organ and hemolymph (Fig. 8) while comparative expression in all the other tissues was relatively low and not significantly different. To confirm this result, a second analysis was carried out with the raw LSNV copy numbers normalized according to the mean *β*-actin copy number for each shrimp and tissue, followed by further analysis of mean relative copy indices as done above based on the non-normalized LSNV copy indices. The result of the second analysis (not shown) was the same as the first, with the highest expression in gill tissue, followed by the lymphoid organ and hemolymph and relatively low expression in the remaining tissues, with significant differences the same as those shown in the graph in Fig. 8. This analysis showed that field tests based on 100 ng template RNA derived from shrimp gill tissue could give an good indication of the severity of LSNV infections without the necessity of concomitant measurement of expression of a host mRNA. This would not be the case for accurate comparison of LSNV levels in time-course laboratory challenge studies or similar studies.

Our results from real-time RT-LAMP analysis for LSNV quantity in various tissues corresponded with previously reported results from *in situ* hybridization assays, where positive signals for LSNV were reported from the cytoplasm of cells in lymphoid organ (LO), gills, haemocytes, brain, eyestalks, optic lobes, ventral nerve cord, cardiac tissue and connective tissue of the hepatopanceas [1], [5], [6]. However, those results were qualitative rather than quantitative and did not clearly indicate tissue preference. Our very low results for hepatopancreatic tissue may have resulted because the *in situ* hybridization results were reported for connective tissue of the hepatopancreas, most of which is located in its sheath, and it is probable that little or no sheath tissue was included in the hepatopancreatic tissue used to make RNA extracts in our study.

Use of our assay protocol to assess viral loads in individual tissues of shrimp infected with LSNV clearly revealed the advantage of quantitative real-time RT-LAMP over conventional non-quantitative LAMP methods. We were able to show that shrimp gill tissue had the highest LSNV loads (p≥0.05) and that they were approximately 2 times higher than those for the next most heavily infected tissue (i.e., lymphoid organ). This is the first time that quantitative tissue loads for LSNV have been reported. The result is important in terms of monitoring and detection of LSNV infections because it reveals that gills should always be used as the target tissue for preparation of RNA templates. This is fortunate, because sufficient gill tissue for analysis can usually be removed without causing undue shrimp stress or mortality. By contrast, sampling lymphoid organ tissue requires killing the shrimp, an option not possible with specimens such as broodstock. The only other suitable tissue for non-destructive sampling is hemolymph, but the level of LSNV in that tissue was approximately 3 times lower than that in gills.

### Advantages of the LSNV real-time RT-LAMP method

This is the first report of quantitative detection of LSNV using a real-time RT-LAMP assay. It proved to be superior to previously reported methods based on nucleic acid amplification techniques (NAAT) such as conventional RT-PCR [1], [8] that are often less sensitive and often do not give a clear indication of severity of infection. Although quantification of LSNV has been reported by real-time RT-PCR assays using SYBR chemistry [25], the method requires sophisticated and expensive equipment that restricts its wide application. Recently, a real-time LAMP method was used to amplify lamda DNA as a model with high specificity and sensitivity by measuring the turbidity that arises from the magnesium pyrophosphate product of the LAMP reaction [10]. There is also increasing interest in simple, rapid and user-friendly turbidimeter detection systems for early and precise diagnosis of human and animal pathogens [13]–[15].

Use of a relatively inexpensive, portable, integrated heating-block/turbidimeter for rapid and sensitive detection of LSNV together with indication of severity of infection by real-time RT-LAMP should allow for wider application of its molecular detection, especially in field laboratories. This is because the approximate estimated cost of the heating-block/turbidimeter at under $3000 US dollars (converted from Thai baht), which is very low when compared to that for a real-time RT-PCR thermocycler. In addition, our LAMP method employs no labeled nucleic acids or special probes that can add to reagent costs for each assay. Further, the RT-LAMP/turbidimeter platform could be adapted relatively easily for detection of other shrimp, veterinary or human pathogens by rational primer design and optimization of the appropriate, specific protocols.
